# Factors associated with long‐term function in cats treated with femoral head and neck excision

**DOI:** 10.1111/jsap.13877

**Published:** 2025-05-07

**Authors:** L. Norberg, C. McGowan, A. Essner

**Affiliations:** ^1^ School of Veterinary Science University of Liverpool Neston UK; ^2^ Blå Stjärnans Djursjukhus Gothenburg Sweden; ^3^ Evidensia Djurkliniken Gefle Gävle Sweden; ^4^ Department of Women's and Children's Health Uppsala University Uppsala Sweden

## Abstract

**Objectives:**

To assess long‐term function in cats following femoral head and neck excision and to investigate if age, weight, sex, breed, outdoor access, multiple injuries, bilateral surgery, complications or physiotherapeutic treatment is associated with long‐term function.

**Materials and Methods:**

Cats treated with femoral head and neck excision without any postoperative major injury or disease were included in a case–control study. An owner‐completed Feline Musculoskeletal Pain Index was used to assess function ≥6 months after surgery. Cats with a score of ≥3 were categorized as having functional impairment (cases). Cats with a score of <3 were categorized as having normal function (controls). Cats' medical records were used to collect information about characteristics, postoperative care and complications.

**Results:**

Thirty‐five cats which had undergone uni‐ or bilateral femoral head and neck excision met the inclusion criteria. Scores ranged from −14 to 11 (median 0, interquartile range 3), 23 (66%) of the cats had normal function and 12 (34%) of the cats had impaired function. Owners of 34 cats (97%) reported good or excellent quality of life. There was an association between being female and a score of ≥3 (odds ratio 24.9; 95% confidence interval: 1.2 to 512.6). No other factors were significantly associated with long‐term function.

**Clinical significance:**

At long‐term follow‐up, owner‐reported quality of life was high. However, 34% of cats had functional impairment and female cats had higher odds of impaired function. This suggests that solely owner‐reported quality of life should not be used to assess outcomes after femoral head and neck excision.

## INTRODUCTION

Femoral head and neck excision (FHNE) is a surgical procedure where the femoral head and neck are removed and a pseudoarthrosis forms (Harper, 2017; Rex, 1963). The purpose of this procedure is to remove the pain arising from the contact between the acetabulum and the femoral head (Krystalli et al., 2023). In cats, FHNE is used to treat diseases or injuries of the coxofemoral joint such as coxofemoral luxations, femoral head and neck fractures, dysplasia and osteoarthritis (Basher et al., 1986; Berzon et al., 1980; Jeffery, 1989; Off & Matis, 2010; Roberts & Meeson, 2022). In comparison to total hip replacement, FHNE is a salvage procedure that is easier and less costly to perform (Schnabl‐Feichter et al., 2021). The owner‐reported convalescence period for cats, the time for return to the cats' normal activities after an FHNE, ranges between 4 and 8 weeks (Off & Matis, 2010; Yap et al., 2015). However, some cats never return to normal activity after this surgery (Berzon et al., 1980; Yap et al., 2015).

High owner‐reported satisfaction and good outcomes of subjective lameness assessment following FHNE have been shown in cats (Off & Matis, 2010; Schnabl‐Feichter et al., 2021; Yap et al., 2015). Although the owners rate the function and quality of life (QoL) of their cats as high, objective measurements have shown deficits in cats' physical ability at long‐term follow‐ups (Liska et al., 2009; Off & Matis, 2010; Schnabl‐Feichter et al., 2021). This included reduced ground reaction force and shortened stance phase duration of the FHNE limb, decreased thigh girth, decreased coxofemoral range of motion (ROM), increased loading on the forelimbs and a displacement of the femur.

How the ostectomy is performed, body weight, initiation of weight‐bearing on the limb, muscle mass and pharmaceutical protocol influence functional outcomes in dogs (Duff & Campbell, 1978; Grisneaux et al., 2003; Montgomery et al., 1987; Off & Matis, 2010). Early weight‐bearing, acute injuries, retention of muscle mass and low body weight have been associated with successful return to function in dogs (Fattahian et al., 2012; Grisneaux et al., 2003; Krystalli et al., 2023; Montgomery et al., 1987; Off & Matis, 2010; Planté et al., 1997). Preservation of the lesser trochanter and incomplete resection have been found to alter functional outcomes in dogs but not in cats (Duff & Campbell, 1978; Grisneaux et al., 2003; Off & Matis, 2010; Schnabl‐Feichter et al., 2021). As factors that influence dogs' function are not consistent with those that influence cats' function, information from studies on dogs should be cautiously transferred to cats. In addition, studies of factors influencing cats post FHNE are scarce. The only factors that have been found to influence cats function post FHNE are a caudodosal displacement of the femur and an increased age at surgery (Schnabl‐Feichter et al., 2021). Both of these factors are associated with asymmetric movements of the forelimbs, which could be a compensatory mechanism to maintain equilibrium and balance control after FHNE (Horak & MacPherson, 1996; Schnabl‐Feichter et al., 2021).

The objective of this study was to assess the long‐term function of cats surgically treated with FHNE using an owner questionnaire. A further objective was to investigate the relationship between the cat's function after FHNE and age, sex, breed, lifestyle, injury, bilateral FHNE, complications and postoperative physiotherapy. The hypotheses were that there would be a positive association between cats' function and lower age at surgery, lifestyle with outdoor access, male sex, early initiation of physiotherapy and implementation of both active and passive physiotherapeutic modalities. The second hypothesis was that there would be a negative association between cats' function and postoperative complications, increased body weight and concurrent injuries.

## MATERIALS AND METHODS

### Study design and inclusion criteria

Ethical approval for this study was granted by the University of Liverpool (reference: VREC1455, date: February 22, 2024).

The study was designed as a case–control study. Inclusion criteria were cats treated with FHNE whose owners were able to read Swedish. A minimum of 6 months was required between the surgery and the completion of the questionnaire. If the cat had undergone FHNE bilaterally, the most recent of the surgeries was assessed. Exclusion criteria were cats that were deceased or that had developed new major injuries or diseases that affected the cat's ability to move. Major injuries were defined as injury to the nervous system, fractures, muscle ruptures or injury to a ligament or tendon.

Owners of cats that had undergone FHNE at Blue Star Animal Hospital, Gothenburg, before July 2023, were contacted. Owners with incomplete contact information or whose cats were marked as deceased were not invited. Advertisements were published (February 26, 2024, and February 29, 2024) on Instagram and Facebook to invite owners whose cats have had an FHNE elsewhere in Sweden.

### Questionnaire and Feline Musculoskeletal Pain Index

The questionnaire was divided into two parts, an initial questionnaire with questions regarding where the cat had been treated and about the cat's current lifestyle, analgesic treatment and injuries/diseases which occurred after the FHNE. Lifestyles were categorised into indoor cats; cats that live indoors and only are outdoors during supervision (in harness or enclosed areas), and outdoor cats; cats that are outdoors without supervision. The questionnaire, translated into English, can be found in the supplementary items (Appendix A1).

The second part of the questionnaire, used to measure the cats' function, was the Swedish translation of the long version of the Feline Musculoskeletal Pain Index (FMPI) questionnaire (Stadig et al., 2019). The FMPI consists of 21 questions divided into three different sections (Benito et al., 2013; Stadig et al., 2019). Activity (questions 1 to 18), pain (questions 19 to 20) and QoL (question 21). The activity questions are scored from left to right; above normal (−1), normal (0), not quite normal (1), somewhat worse than normal (2), barely or with great effort (3) and not at all (4), the answer ‘does not apply or I don't know’ are not being scored. The pain questions were scored; no pain (0), little pain (1), mild pain (2), moderate pain (3) and severe pain (4). Quality of life was scored; excellent, good, fair or poor.

### Medical records

Medical records were reviewed for all cats whose owners completed the questionnaire. Data extracted from the medical records included the following: date of birth, breed, sex, body weight, outdoor access before surgery, date of surgery, weight at surgery, type of injury, side of FHNE, postoperative complications and postoperative physiotherapy.

The type of injury was divided into three subgroups: fracture of the femur, coxofemoral luxation and multiple injuries. Postoperative physiotherapy was divided into initiation of physiotherapy and physiotherapy modalities used. Initiation of physiotherapy was recorded as days between surgery and the first physiotherapeutic intervention recorded in the medical records. Physiotherapy modalities were divided into passive modalities (e.g., massage, stretching, passive ROM exercises and low light laser therapy) and active physiotherapy (all exercises where the cat did an active movement, e.g., balancing, muscle strengthening and active ROM exercises).

### Statistical analyses

A priori sample size calculations were carried out using Epitools (Sergeant, 2018). A sample of 40 cats (20/group) was calculated to be required to have 80% power to detect odds ratios (OR) of 6, with a 95% level of confidence.

Analysis was done using IBM SPSS statistics (version 29.0.1.0). Questions 1 to 20 of the FMPI were used to calculate a total score and a cut‐off value of 3 was used as proposed by Stadig et al. (2019). Cats were classified as cases if FMPI score ≥3 or controls if FMPI score <3. All data were non‐normally distributed. Age, time since surgery, weight and initiation of physiotherapy were presented with median and interquartile range (IQR). A Mann–Whitney *U* test was used to assess if the numerical data (weight, age at surgery, time since surgery and initiation of physiotherapy) were associated with either cases or controls. A Fisher's Exact Test was performed for analysing the association between categorical data (breed, sex, lifestyle, multiple injuries, bilateral FHNE, complications and active physiotherapy) and the function of case or control. A Fisher's Exact Test was used when the categorical data were analysed. When calculating OR for sex, one of the cells contained zero cats; a Haldane‐Anscombe correction (adding 0.5 to each cell) was performed manually to obtain a calculation of the OR and its 95% confidence intervals (CI) (Weber et al., 2020). The significance level was set at P < 0.05.

## RESULTS

### Descriptive data

In total, 35 (31 male, 4 female) cats were included in the study (Table 1). Median age at follow‐up was 55 (IQR 53) months. Breeds included domestic short‐/long‐haired, mixed breeds, Maine coon, Siberian, Neva Masquerade, Birman, Bengal and Norwegian forest cat. The majority of cats, 21 (60%) were domestic short‐ or long‐haired cats; 12 (34%) of the cats were pedigree and two cats (6%) were mixed breed.

**Table 1:** Description of the population of cats (*n* = 35) in the study of long‐term level of function following femoral head and neck excision surgery (FHNE). Categorical variables presented as frequencies and proportions (%)

		Overall	Cases	Controls
Age at surgery (months)	Median	18	18.5	18
	Range	5–96	11–30	5–96
Time since surgery (months)	Median	31	37	30
	Range	14–121	21–119	14–121
Age at follow up (months)	Median	55	54	57
	Range	32–164	33–134	32–164
Sex	Male	31 (88.6)	8 (66.7)	23 (100.0)
	Female	4 (11.4)	4 (33.3)	0 (0.0)
Breed	DSH/DLH^†^	21 (60.0)	7 (58.3)	14 (60.9)
	Maine coon	6 (17.1)	3 (25.0)	3 (13.0)
	Mixed breed	2 (5.7)	1 (8.3)	1 (4.3)
	Neva Masquerade	2 (5.7)	1 (8.3)	1 (4.3)
	Norwegian forest cat	1 (2.8)	0 (0.0)	1 (4.3)
	Birman	1 (2.8)	0 (0.0)	1 (4.3)
	Bengal	1 (2.8)	0 (0.0)	1 (4.3)
	Siberian	1 (2.8)	0 (0.0)	1 (4.3)
Lifestyle	Outdoor	22 (62.9)	9 (75.0)	13 (56.5)
	Indoor	13 (37.1)	3 (25.0)	10 (43.5)
Weight (kg)	Median	4.8	4.8	4.9
	Range	3.0–8.2	3.5–8.2	3.0–6.8
Injuries	Femoral fracture	21 (60.0)	7 (58.3)	14 (60.9)
	Coxofemoral luxation	8 (22.9)	3 (25.0)	5 (21.7)
	Multiple Injuries	6 (17.1)	2 (16.7)	4 (17.4)
Bilateral FHNE		5 (14.3)	1 (8.3)	4 (17.4)
Complications		8 (22.9)	1 (8.3)	7 (30.4)
Initiation of physiotherapy (days)	Median	1	1	1
	Range	1–14	1–2	1–14
Received postoperative physiotherapy		35 (100.0)	12 (100.0)	23 (100.0)
Underwent active exercises (*n* = 34)		26 (76.5)	10 (83.3)	16 (72.7)
Received analgesia at follow up		0 (0.0)	0 (0.0)	0 (0.0)

^†^ Domestic short haired/Domestic long haired

Five cats (14%) had been treated with FHNE bilaterally (Table 1). Reasons for performing a FHNE were femoral fractures in 21 cats (60%), coxofemoral luxations in eight cats (23%) and due to multiple injuries in six cats (17%). Multiple injuries included at least two of the following injuries: hip dysplasia, lumbosacral disease, luxations of the coxofemoral joint, sacroiliac joint, coccygeal and/or patella and fracture of the ilium, acetabulum, sacrum, os pubic, crista tibia and/or femur. Postoperative complications occurred in eight (23%) of the cats. Complications included oedema (*n* = 2), infection (*n* = 3), pyrexia (*n* = 1), thoracolumbar pain (*n* = 1), caudodorsal displacement (*n* = 1) and medial patella luxation (*n* = 1). At the time for follow‐up questionnaire completion, none of the cats were treated with analgesics and 22 (63%) of the cats had an outdoor lifestyle.

All cats underwent postoperative physiotherapeutic treatment (Table 1). Physiotherapy was initiated within 3 days for 97% of the cats, with a median of 1 (IQR 0) day. Physiotherapy modalities were passive and/or active modalities. One cat had missing data for physiotherapeutic treatment as medical records from treatment post‐hospitalisation were not obtained. This resulted in a smaller sample size (*n* = 34) for the active physiotherapy variable. Active exercises were used for 26 (76%) of the cats.

### Feline Musculoskeletal Pain Index

**FIG 1:**
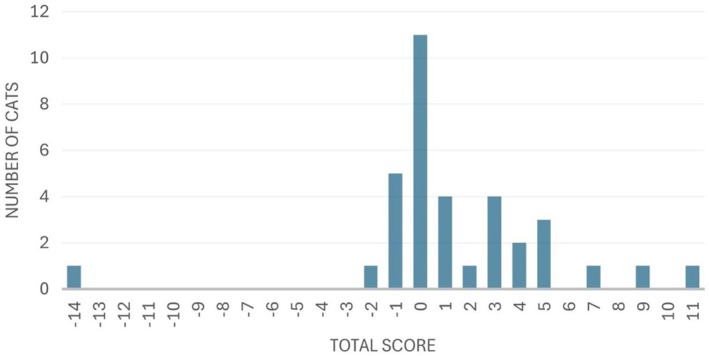
Frequencies of the Feline Musculoskeletal Pain Index (FMPI) scores in cats (*n* = 35) at least 14 months after femoral head and neck excision surgery.

Total FMPI scores for all cats ranged from −14 to 11, with a median of 0 (IQR 3) (Fig 1); 23 (66%) cats had an FMPI score of <3 and were categorised as controls, and 12 (34%) cats had an FMPI score of ≥3 and were categorised as cases.

**FIG 2:**
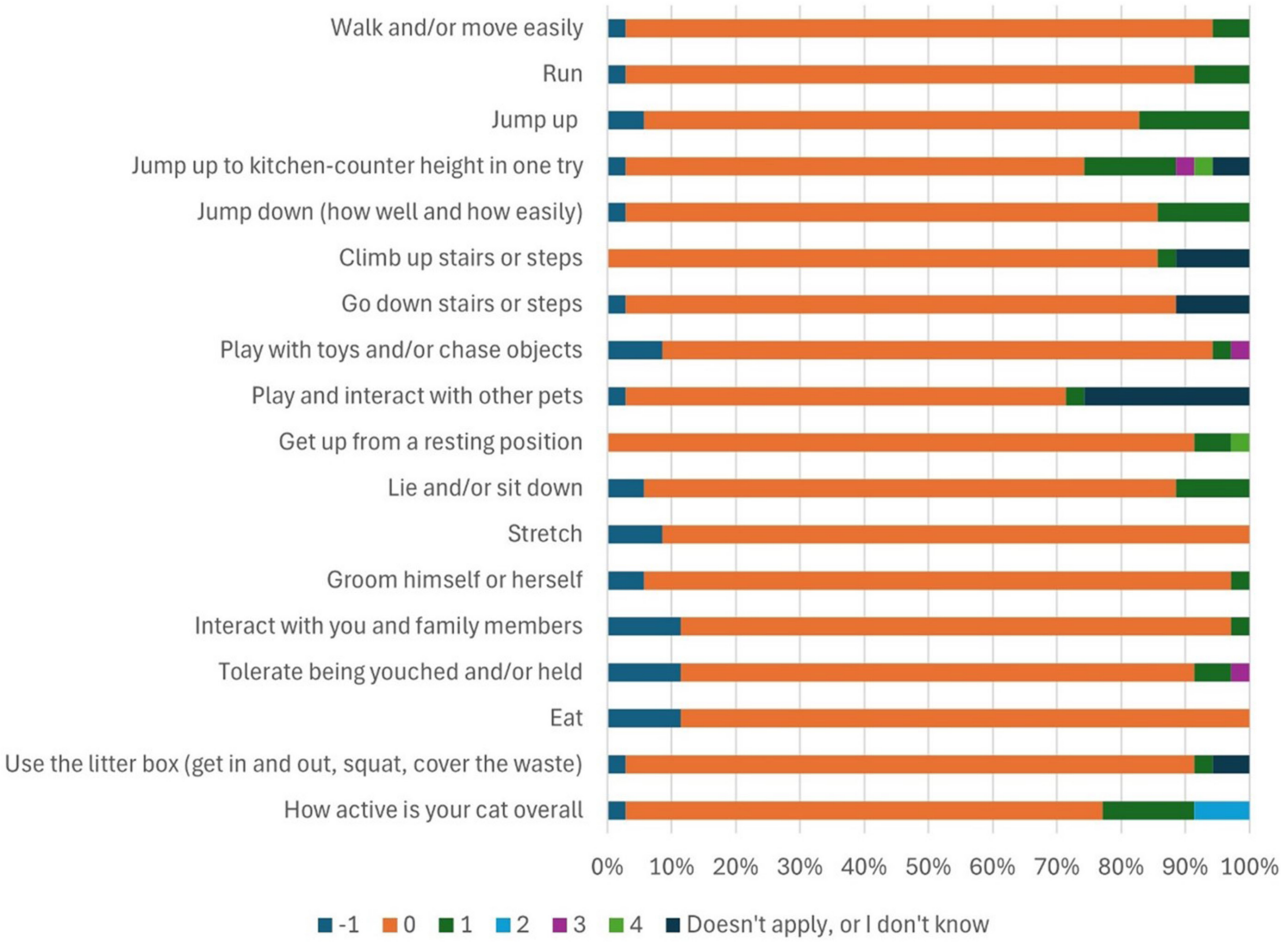
Owner‐reported scores of each of the activity questions, question 1 to 18 of the Feline Musculoskeletal Pain Index. Presented as proportion of all cats (*n* = 35) in a long‐term follow‐up of function at least 14 months after femoral head and neck excision surgery. Scores are in relationship to the question: ‘What can your cat do compared to a normal cat?’ −1 = above normal, 0 = normal, 1 = not quite normal, 2 = somewhat worse than normal, 3 = barely or with great effort, 4 = not at all, doesn't apply or I don't know.

The three most frequently reported activities where cats in this study showed impaired function were ‘Jump up’, ‘Jump up to kitchen counter height in one try’ and ‘How active is your cat overall’ (Fig 2). Six (17%) of the cats were reported to be ‘not quite normal’ when jumping up, seven (20%) of the cats had various difficulties jumping up to the kitchen counter in one try and eight (23%) of the cats were reported to have reduced activity. In female cats, one (25%) cat was reported to have a decreased ability to jump up to kitchen counter height in one try, and none of the female cats were reported to have difficulties jumping up. The two categories where female cats were reported to have an impaired function were, ‘Handle being touched and/or held’ (two cats, 50%) and ‘How active is your cat’ (two cats, 50%).

Overall, 29 cats (83%) were reported to have no pain, and six cats (17%) were reported to have pain over the last week; five of these cats were reported to have little pain, and one was reported to have moderate pain. Thirty (86%) of the cats were reported to have no pain, and five (14%) of the cats were reported to have pain today; four of the cats were reported to have little pain, and one of the cats had moderate pain. In females, three (75%) of the cats were reported to have pain both the past week and today; two of the cats had little pain, and one had moderate pain.

Owners of 26 (74%) cats reported an excellent QoL, 8 (23%) owners reported a good QoL, and 1 (3%) owner reported a fair QoL. The cat reported to have a fair QoL was a male cat.

### Association between factors and function

There was no significant difference between the groups regarding age at surgery, time since surgery, body weight at surgery and initiation of physiotherapy. Median age at surgery was 18.5 (IQR 9) months for cases and 18 (IQR 10) months for controls (P = 0.53). The median time since surgery was 37 (IQR 45) months for cases and 30 (IQR 61) months for controls (P = 0.42). Median body weight at surgery was 4815 (IQR 2835) grams for cases and 4970 (IQR 1230) grams for controls (P = 0.81). The median time for initiation of physiotherapy was 1 day (IQR 0) for cases and 1 day (IQR 0) for controls (P = 0.44).

Females had increased odds of an FMPI score of ≥3 (OR: 24.9; 95% CI: 1.2 to 512.6; P = 0.009). Breed (OR:0.9; 95% CI: 0.2 to 4.1; P = 1.00), lifestyle (OR: 0.4; 95% CI: 0.1 to 2.0; P = 0.46), multiple injuries (OR:1.0; 95% CI: 0.1 to 6.1; P = 1.00), bilateral FHNE (OR:0.4; 95% CI: 0.04 to 4.4; P = 0.64), postoperative complications (OR:0.2; 95% CI: 0.2 to 1.9; P = 0.22) and active physiotherapy exercises (OR:1.9; 95% CI: 0.3 to 11.2; P = 0.68) were not significantly associated with long‐term function.

## DISCUSSION

This study showed that the long‐term function was normal in 66% and decreased in 34% in cats treated with uni‐ or bilateral FHNE. The decreased physical ability in 34% of the cats at long‐term follow‐up is accordant with previous studies which found that 39% of the cats had a reduced jump height (Yap et al., 2015). Activity monitoring has found that both the activity and jump frequency are reduced in older cats (Yamazaki et al., 2020). This should however not impact the result in this study as the median age was 55 months. A previous study has found that owners report a high QoL post‐FHNE (Yap et al., 2015). This is accordant with this study where 97% of the owners reported a good or excellent QoL. In both this study and earlier studies, the owner‐reported QoL did not reflect outcomes from validated measurements (Off & Matis, 2010; Schnabl‐Feichter et al., 2021). There could be different reasons for this. Signs of pain can be subtle; a previous study on cats with cranial cruciate ligament injuries found that a large proportion of the cats had FMPI scores which indicated pain but that owners did not perceive the cat as painful (Boge et al., 2020). This indicates that chronic pain in cats can be hard to notice. Caregiver placebo could be another reason (Conzemius & Evans, 2012). Hence, owner‐reported QoL should not be used as a single tool to assess outcome after FHNE in cats. However, owner assessment is an important tool to identify behavioural changes in the cat which could indicate pain, as subjective owner‐reported questionnaires have been used with good results, showing that owners could assess changes in their cat's functional impairments related to chronic pain (Bennett & Morton, 2009). The FMPI has been developed to assess behavioural changes and pain in cats and has been found to have a good to excellent internal consistency, reliability, repeatability, discriminatory ability and validity (Benito et al., 2013; Enomoto et al., 2022; Stadig et al., 2019).

In this study, an association was found between female cats and a decreased function at long‐term follow‐up. In the current study, female cats had poorer FMPI scores compared to male cats. This result might have been biased because of the small sample size of female cats overall and that none of the cats in the controls were females. A previous study that used veterinary data to assess the prevalence of common disorders in cats found that female cats had a higher prevalence of poor QoL compared to male cats (O'Neill et al., 2023). There could be an association between decreased function and poor QoL, however, all of the female cats in the current study were reported to have a good QoL. It has been found that dogs with a lively temperament and live with owners who promote activity have better outcomes post‐FHNE (Planté et al., 1997). A possible hypothesis for the sex difference in FMPI scores was, therefore, that activity levels might differ between sexes and that a lower activity level might result in a decreased long‐term function. However, activity monitoring measuring activity levels in cats has not shown a difference in activity levels between the sexes (Yamazaki et al., 2020). Another hypothesis related to activity level was that cats with outdoor access would have better long‐term function compared to cats that were not allowed to be outdoors unsupervised. There was, however, no significant association between outdoor access and function in this study. Maniaki et al. (2021) found that cats with outdoor access have an increased risk of degenerative joint disease at the age of six. The FMPI has been found to discriminate between cats with or without early signs of degenerative joint disease (Maniaki et al., 2023). In this study, no physical examination of the cats was performed at the time of questionnaire completion. Hence, it cannot be ruled out that some of the cats had early degenerative joint diseases in other joints that had not been detected yet.

Complications reported in this study were not associated with the cats' physical function in daily activities. This is accordant with earlier studies that did not find any association between caudodorsal malposition and lameness in cats; or oedema, infection or fever with medium‐term functional outcome in dogs (Lewis et al., 1988; Schnabl‐Feichter et al., 2021). The cat with thoracolumbar pain was the only cat with complications that had an impaired function at long‐term follow‐up. The thoracolumbar pain could have affected the FMPI scoring of the cat as thoracolumbar pain might affect the cat's behaviour. After FHNE, physiotherapy should be initiated early to promote weight‐bearing, coxofemoral ROM and improve muscle function (Berzon et al., 1980). Physiotherapy is frequently recommended post‐FHNE; however, physiotherapeutic protocols differ between studies (Colvero et al., 2020; Dycus et al., 2017; Harper, 2017; Sabiza et al., 2019). In this study, all cats received postoperative physiotherapy. About a quarter of the cats received passive physiotherapeutic modalities such as passive ROM exercises, massage, stretching and low laser therapy, while three out of four cats participated in active therapeutic exercises as well. Active therapeutic exercises, in addition to the ROM exercises, were hypothesised to result in a better function as great muscle mass is a positive factor in dogs (Montgomery et al., 1987; Off & Matis, 2010). However, no association was found between active therapeutic exercises and long‐term function in this study. Early initiation of physiotherapy was hypothesised to be beneficial to treat several postoperative problems including muscle atrophy, reduced weight bearing and reduced ROM. Muscle atrophy occurs as early as 3 days postoperatively, where early physiotherapy can attenuate postoperative muscle atrophy; early postoperative weight‐bearing decreases the time to final weight‐bearing after FHNE, and ROM exercises form the flexibility of the pseudoarthrosis and decrease postoperative pain (Baltzer, 2020; Krystalli et al., 2023; Millis et al., 2004).

There are several limitations in this study. As no physical examination was performed, it cannot be excluded that the cats could have had an undetected concurrent injury or disease which could have affected the FMPI scores. In addition, case–control studies are reported to be susceptible to bias (Schulz & Grimes, 2002). In this study, there is a risk that misclassification bias might have been inadvertently introduced as the hypothesis was not blinded for the authors before data collection. Nonparticipation and the small sample size might have induced selection bias, and the small sample size may also have resulted in a Type II error where significant effects were missed.

In conclusion, after an average of 31 months post FHNE surgery, the owner reported QoL was high; however, one third of the cats had functional impairments. The results in the present study indicate that female cats have higher odds for long‐term functional impairments following FHNE. No other risk or protective factors were detected.

## Conflict of interest

None of the authors of this article has a financial or personal relationship with other people or organisations that could inappropriately influence or bias the content of the paper.

## Author contributions

**L. Norberg:** Conceptualization (equal); data curation (lead); formal analysis (lead); investigation (lead); methodology (equal); project administration (lead); resources (lead); validation (equal); visualization (equal); writing – original draft (lead); writing – review and editing (lead). **C. McGowan:** Conceptualization (equal); methodology (equal); supervision (equal); validation (equal); writing – review and editing (equal). **A. Essner:** Conceptualization (equal); methodology (equal); supervision (equal); validation (equal); visualization (equal); writing – review and editing (equal).

## Supporting information


Appendix S1.

## Data Availability

The data that support the findings of this study are available from the corresponding authors, upon reasonable request.
