# Supplementary material for: Factors associated with long‐term function in cats treated with femoral head and neck excision
Source: J Small Anim Pract. 2025 May 7;66(10):693–9. doi: 10.1111/jsap.13877 (PMC12489522; doi:10.1111/jsap.13877)
Supplement: Supplementary file 1 — Appendix S1. [file JSAP-66-693-s001.docx]

**Appendix A: Questionnaire (translated into English)**

A questionnaire about the cat’s ability to perform daily activities – at least six months after the ball has been removed from the hip ball-and-socket joint

Information about you and your cat

1. Your full name: _______________________

** To be able to find the cat's medical journal you must be the owner of the cat*

2. Name of the cat:_________________________

3. The cat´s date of birth: YY-MM-DD

4. Has your cat had another injury or disease, which affects the cat’s mobility, after the removal of the ball of the hip joint?

- YES - injury *(the participant will go to the next question)*
- YES – disease *(the participant will jump to question 6)*
- NO *(the participant will jump to question 7)*

5. If Yes - injury:

What type of injury did your cat have?

- Fracture *(the participant will go to the end of the form)*
- Injury to the brain, to a nerve or the spinal cord *(the participant will go to the end of the form)*
- Injury to a ligament or tendon *(the participant will go to the end of the form)*
- Rupture of a muscle *(the participant will go to the end of the form)*
- Other: __________ *(the participant will go to question 7)*

6. If Yes – disease:

What disease is your cat affected from? __________________________________

7. Does your cat get any pain medication?

- YES
- NO

8. What is your cat’s lifestyle?

**Outdoor means that your cat has the opportunity to go outside freely without supervision. A cat that is only indoors or that is outside only on a leash or within a fenced area is counted as an indoor cat.*

- INDOOR
- OUTDOOR

9. At which clinic did your cat remove the ball of the hip joint? __________________________________

10. At which clinic did your cat do the follow-ups (for example visits to a physiotherapist or veterinarian)?
